# The Efficacy of Phage Therapy in a Murine Model of *Pseudomonas aeruginosa* Pneumonia and Sepsis

**DOI:** 10.3389/fmicb.2021.682255

**Published:** 2021-07-05

**Authors:** Xu Yang, Anwarul Haque, Shigenobu Matsuzaki, Tetsuya Matsumoto, Shigeki Nakamura

**Affiliations:** ^1^Department of Microbiology, Tokyo Medical University, Tokyo, Japan; ^2^Department of Infectious Diseases, School of Medicine, International University of Health and Welfare, Narita, Japan; ^3^Department of Medical Laboratory Science, Kochi Gakuen University, Kochi, Japan

**Keywords:** *Pseudomonas aeruginosa*, pneumonia, sepsis, bacteriophage, phage therapy

## Abstract

The emergence of multi-drug resistant *Pseudomonas aeruginosa* necessitates the search for treatment options other than antibiotic use. The use of bacteriophages is currently being considered as an alternative to antibiotics for the treatment of bacterial infections. A number of bacteriophages were introduced to treat pneumonia in past reports. However, there are still lack of knowledge regarding the dosages, application time, mechanism and safety of phage therapy against *P. aeruginosa* pneumonia. We used the bacteriophage KPP10 against *P. aeruginosa* strain D4-induced pneumonia mouse models and observed their outcomes in comparison to control models. We found that the nasal inhalation of highly concentrated KPP10 (MOI = 80) significantly improved survival rate in pneumonia models (*P* < 0.01). The number of viable bacteria in both lungs and in serum were significantly decreased (*P* < 0.01) in phage-treated mice in comparison to the control mice. Pathological examination showed that phage-treated group had significantly reduced bleeding, inflammatory cell infiltration, and mucus secretion in lung interstitium. We also measured inflammatory cytokine levels in the serum and lung homogenates of mice. In phage-treated models, serum TNFα, IL-1β, and IFN-γ levels were significantly lower (*P* < 0.05, *P* < 0.01, and *P* < 0.05, respectively) than those in the control models. In the lung homogenate, the mean IL-1β level in phage-treated models was significantly lower (*P* < 0.05) than that of the control group. We confirmed the presence of phage in blood and lungs, and evaluated the safety of bacteriophage use in living models since bacteriophage mediated bacterial lysis arise concern of endotoxic shock. The study results suggest that phage therapy can potentially be used in treating lung infections caused by *Pseudomonas aeruginosa*.

## Introduction

*Pseudomonas aeruginosa* is a gram-negative opportunistic pathogen and is one of the main pathogens that cause nosocomial infections. It is a common etiology for infections in immunocompromised patients (Parker et al., 2008; Gellatly and Hancock, 2013) and in respiratory-associated pneumonia in ICU settings. Antimicrobial therapy is the choice of treatment in *Pseudomonas* infections; however, due to the emergence of multi-drug resistant *P. aeruginosa*, there have been cases wherein antibiotics have failed (Nathwani et al., 2014; Jernigan et al., 2020). Phage therapy is considered as a treatment option against bacterial infections because of its ability to lyse bacterial cells. Compared to antimicrobial therapy, phage therapy has advantages such as high specificity, sterilization, self-propagation, and protection of mutation-mediated antibiotic-resistance development (Matsuzaki et al., 2005). In recent years, due to the emergence of multi-drug resistant bacteria, phage therapy has attracted widespread attention due to its possession of a completely different mechanism of action compared to antibiotic therapy (Viertel et al., 2014).

Although phage therapy has a long history, the safety of phage therapy has not yet been fully studied (Caflisch et al., 2019; Kwiatek et al., 2020) especially in the context of infections caused by gram-negative bacteria that pose a risk of endotoxemia (Buttenschoen et al., 2010; Dickson and Lehmann, 2019). Endotoxins are present in the cell walls of most gram-negative bacteria, and even very low doses can strongly trigger human inflammatory reactions, and in severe cases can cause endotoxin shock and death (Lepper et al., 2002; Surbatovic et al., 2013). Like bactericidal antibiotics, bacteriophage causes release of endotoxins when they lyse gram-negative bacteria (Dufour et al., 2017). Past reports showed that phage therapy was used in children to treat persistent lung infections with cystic fibrosis caused by *P. aeruginosa* (Krylov et al., 2016; Parkins et al., 2018); however, there are several reports of pneumonia with *P. aeruginosa* in patients with non-cystic fibrosis (Patey et al., 2019). Therefore, we aimed to evaluate the therapeutic effect of phage therapy on pulmonary *P. aeruginosa* infection using animal models.

## Materials and Methods

### Bacteria Strain

The *P. aeruginosa* standard strain D4 was isolated from the blood of neutropenic mice which had bacteremia (Matsumoto et al., 1997a,b, 1998a,b,c; Watanabe et al., 2007); this was stored in LB medium containing 50% glycerol at −80°C.

### Animal Care and Use

Six weeks old Male, pathogen-free, ICR mice that were purchased (Sankyo Labo Service Co., Ltd.) and were housed under specific-pathogen-free conditions and were supplemented with standard laboratory food and water *ad libitum*. The facility was maintained at a constant temperature (27°C), humidity (65%), and 12/12 h light/dark cycle.

### Anesthesia Protocol

The anesthesia mixture used was prepared by combining the following: 2 mL midazolam (5 mg/mL; FUJIFILM Wako Co., Ltd.), Vetorphale 2.5 mL (5 mg/mL; Meiji Seika Pharma Co., Ltd.), medetomidine hydrochloride 0.75 mL (1 mg/ml; Kyoritsu Seiyaku), normal saline 19.75 mL (Otsuka Pharmaceutical Co., Ltd.). The mice were anesthetized by subcutaneously injecting the anesthesia mixer at a dose of 0.1 mL/10 g. The animal study was reviewed and approved by Tokyo Medical University Animal Care and Use Committee.

### Phage

Using *P. aeruginosa* strain (P20) as the host, bacteriophage strain KPP10 was isolated from a water sample collected from a river in Kochi Prefecture, Japan. Strain P20 was derived from clinical specimens of Kochi Medical University Hospital in Kochi Prefecture, Japan. The KPP10 bacteriophage belongs to the Myoviridae family, morphological type A1, and has strong lytic activity against *P. aeruginosa* D4 (Watanabe et al., 2007; Uchiyama et al., 2012). Previous reports indicate that bacteriophage KPP10 is a lytic bacteriophage, which has no pathogenicity or pathogenicity-related genes, so it is expected to be very suitable for use as a therapeutic bacteriophage (Uchiyama et al., 2012).

### Bacteriophage Culture

LB medium was inoculated with 1% v/v of overnight bacterial culture at the exponential growth phase and was incubated at 37°C for 2 h to reach an approximate OD_600_ of 0.2–0.4. Then, the phage was added at MOI = 10 and was incubated at 37°C for 5 h with gentle stirring (100 rpm) to facilitate cell lysis. Cell debris was removed by centrifugation (20 min, 1,750 × *g*, 4°C). The supernatant was filtered using a 0.22 μm disposable filter (CORNING 430521) and the phage were stored at 4°C for downstream use.

### Phage Count

The phages were counted using the plaque assay method according to standard protocols (Parker et al., 2008). Phage dilutions were prepared using physiological saline. Using 2% agar as the bottom layer, 20 mL of LB agar medium (Sigma) containing 100 μL of phage dilution and 100 μL of exponentially growing bacterial solution were plated on agar plates at 45°C. The plate was then incubated at 37°C overnight, afterward, the plaques were counted.

### PEG Precipitation

The bacteriophage was mixed with polyethylene glycol (PEG) 6000 [10% total (wt/vol); FUJIFILM Wako] and NaCl (0.5 M; FUJIFILM Wako), and were kept in rotation overnight at 4°C. Then, these were centrifuged for 15 min at a speed of 11,000 × *g* in 4°C (TOMY RX200) to collect the precipitate, and pellet was suspended in phage buffer to dilute the phage concentration 200 times. We noticed that the use of ultracentrifugation to concentrate the phage solution led to ammonium acetate and cesium chloride release, which proved to be toxic. As such, we used high-speed centrifugation in the presence of PEG precipitation. In the method described by Bourdin et al. (2014) chloroform was used to remove bacterial proteins in the concentrate after centrifugation; however, KPP10 is not chloroform-resistant (data not shown), so this step was omitted.

### *Pseudomonas aeruginosa* Pneumonia Model

*Pseudomonas aeruginosa* D4 cultured on LB agar plates overnight at 37°C was adjusted to 2.5 × 10^9^ colony forming unit (CFU)/mL using sterile physiological saline. To produce the *P. aeruginosa* pneumonia model, 20 μl of bacterial suspension was inoculated through intranasal route in anesthetized mice. These mice were randomly divided in two groups and 10 μL of KPP10 bacteriophage (4 × 10^11^/mL plaque forming unit, PFU) (MOI = 80) was administered in these mice intranasally after 2 h (group 1) and 8 h (group 2) of bacterial inoculation. Past study suggested that higher dose (MOI) of bacteriophage can promptly kill bacteria and help to minimize the chance of resistant development to phage (Taha et al., 2018). In addition, another study using in-vitro models showed that KPP10 became resistant to *P. aeruginosa* D4 strains at 210 min. of administration (Watanabe et al., 2007). Considering number and short therapeutic window of phage from these studies, we administered the bacteriophage in mice at a highest concentration (MOI) that we could harvest in our laboratory (MOI = 80). The mice in negative control (NC) group were inhaled 10 μL of phage buffer after 2 h of inoculation. We used imipenem/cilastatin sodium to prepare a positive control (PC) group. After 2 h post inoculation of *P. aeruginosa*, 25 mg/kg of imipenem/cilastatin sodium (MSD Co., Ltd.) was injected subcutaneously to a group of randomly selected mice at every 12 h for 3 days (Coopersmith et al., 2003). All groups were monitored daily for 6 days to calculate their survival rate.

Analysis of survival data showed that there was no significant difference in survival rates between the mice those inhaled KPP10 at 2 h and 8 h p.i. Therefore, in the bacteriology and histopathology experiments we only included the Pa pneumonia mice inhaled either phage KPP10 or phage buffer at 2 h p.i.

### Pathological Examination of Lung Tissue

After 2 h of inhalation of 20 μL *P. aeruginosa* D4 (1.25 × 10^9^/mL CFU), the treatment group inhaled 10 μL phage KPP10 (2 × 10^11^/mL PFU), and the control group inhaled 10 μL of sterile phage buffer. After 24 h, mice were euthanized by isoflurane inhalation. The lungs and trachea were removed under sterile conditions, and the specimens were fixed with 10% formalin (FUJIFILM Wako). The specimen was embedded and sliced, and then stained with H&E stain.

### Determination of Viable Bacteria and Phage in the Lung and Serum

*Pseudomonas aeruginosa pneumonia* mice were randomly divided in treatment and control groups. After 2 h post infection, the treatment group inhaled 10 μL (MOI = 80) of phage KPP10 (2 × 10^11^ PFU/mL), and the control group inhaled 10 μL of phage buffer. After 24 h, live mice were euthanized by isoflurane inhalation to collect their blood from cardiac ventricles and the whole lung under sterile conditions. A tissue homogenizer (Nakayama Co., Ltd., 16–80) was used to homogenize the lung tissues in physiological saline, after which EDTA (final concentration 0.05 M) was added, and the tissue debris were removed by centrifugation at 8,000 × *g* for 2 min at 4°C. Collected lungs were kept on ice during all steps. Blood samples were coagulated in a vacuum blood collection tube (Nipro Corporation Limited), and the serum was separated from blood cells by centrifugation at 10,000 × *g* for 5 min. The serum and lung tissue homogenate samples were diluted with sterile physiological saline and inoculated on NAC agar plates (Eiken Chemical Co., Ltd.), and incubated at 37°C for 24 h.

Serum and lung tissue homogenate samples were prepared using physiological saline. Using 2% agar as the bottom layer, 20 mL of LB agar medium (Sigma) containing 100 μL of samples and 100 μL of exponentially growing bacterial solution were plated on agar plates at 45°C. The plate was then incubated at 37°C overnight, afterward the plaques were counted. Serum and lung tissue homogenate samples were stored at −30°C for downstream analyses.

### Measurement of Cytokine and High Mobility Group Box 1 (HMGB1) Level

TNF-α, IL-1β, and IFN-γ concentrations in serum and lung tissue samples and serum HMGB1 concentration were assayed using mouse uncoated ELISA Kit (Thermo Fisher Scientific, MA, United States) and mouse/rat HMGB1 ELISA Kit (Arigo Biolaboratories Co., Ltd., Hsinchu City, Taiwan) according to the manufacturer’s protocols, respectively.

### Determination of Endotoxin Levels in Serum

We used a commercial limulus amebocyte lysate (LAL) endotoxin detection kit (FUJIFILM Wako) to measure the endotoxin levels of the collected mouse serum samples. Mouse serum samples were diluted 10-fold using physiological saline (Otsuka Pharmaceutical). Then, 100 μL of the diluted serum sample was added to the endotoxin sample pretreatment solution (FUJIFILM Wako), mixed, and incubated at 70°C for 10 min, after which ice water was immediately added to the mixture and to cool for 2 min. Afterward, 200 μL of the treated sample was then added to the LAL reagent, thoroughly mixed, and measurement was done using Toxinometer MT-6500 (FUJIFILM Wako).

### Statistical Analysis

Survival analysis was performed using the chi-square test, and the survival rate by the Kaplan–Meier method. All other assay data are presented as the mean ± standard deviation (SD). Differences between groups were examined using the Mann–Whitney *U* test (*P* values < 0.05 were considered to indicate a statistically significant difference).

## Results

### Survival Rate of *Pseudomonas aeruginosa* Pneumonia Model

**FIGURE 1:**
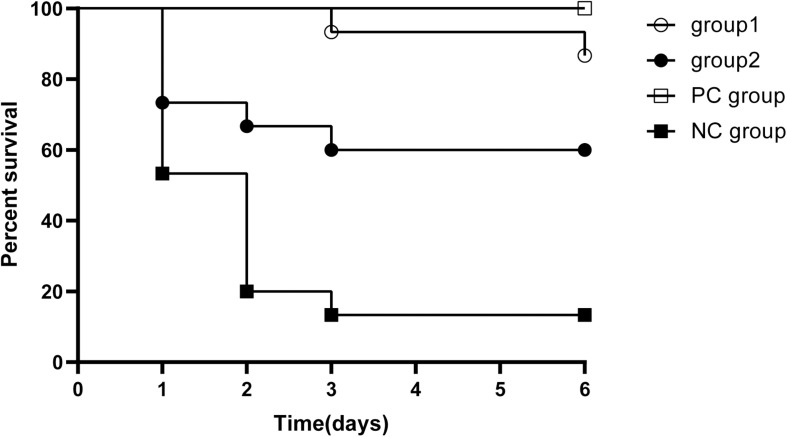
Survival rate in pneumonia mice. All mice inhaled 20 μL *P. aeruginosa* D4 (2.5 × 10^9^ CFU/ml). Group 1 (*n* = 15) inhaled 10 μl phage KPP10 (10 μl 4 × 10^11^ PFU/ml) at 2 h post bacterial inoculation. Group 2 (*n* = 15) inhaled 10 μl phage KPP10 (10 μl 4 × 10^11^ PFU/ml) at 8 h post bacterial inoculation. Negative control (NC) group inhaled 10 μL of phage buffer at 2 h post bacterial inoculation and the positive control (PC) group received subcutaneous injection imipenem/cilastatin sodium (25 mg/kg) at 2 h post infection and at every 12 h interval for 3 days. Survival rate in group 1, group 2 and PC mice were significantly higher (*p* < 0.01) when individually compared with the survival rate of NC mice. CFU denotes colony forming unit. PFU denotes plaque forming unit.

In order to evaluate whether KPP10 has a protective effect against pneumonia caused by *P. aeruginosa* D4, we compared survival rates between four groups (Figure 1). The survival rate of mice in the group 1, group 2 and the PC group were significantly higher when individually compared with that of the NC mice (87% vs. 13%; *p* < 0.01, 60% vs. 13%; *p* < 0.01, 100% vs. 13%; *p* < 0.01). The survival rate between group 1 and group 2 and between group 1 mice and PC group had no significant differences (87% vs. 60%; *p* = 0.99, 87 vs. 100%; *p* = 0.143). This indicates that KPP10 has a protective effect against pneumonia caused by *P. aeruginosa* D4. Moreover, even with an administration of phage at a delayed time point of post infections, survival rate in pneumonic mice improved.

### Pathological Examination

**FIGURE 2:**
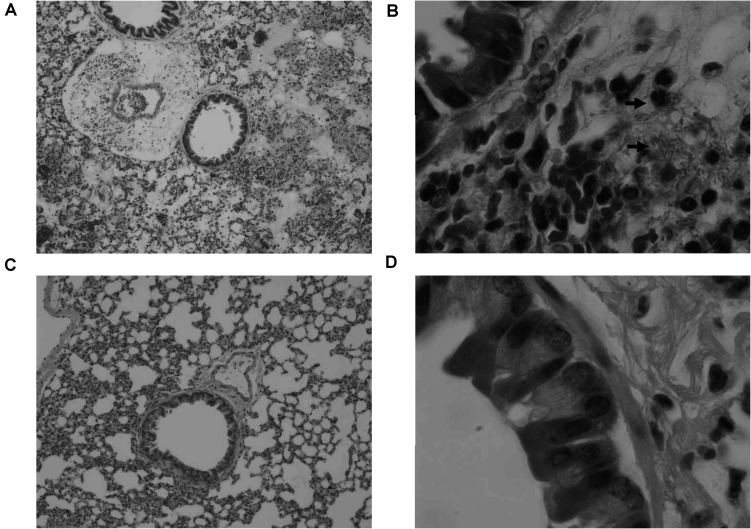
Pathological micrograph of mouse lungs. In the control group (*n* = 6), 50× magnification **(A)** showed that the alveoli were filled with red blood cells and mucus, and a tendency of severe pulmonary bleeding was observed. At 1,000 × magnification **(B)**, there are a large number of bacteria in the lungs, and inflammatory cell infiltrations in the lung interstitium can be seen. In the treatment group (*n* = 6), a small amount of red blood cells in the alveoli can be seen at 50 × magnification **(C)**. At 1,000 × magnification **(D)**, the infiltration of inflammatory cells can be seen whereas no bacteria can be found.

In order to confirm the effect of KPP10 on pneumonia caused by *P. aeruginosa* D4, we observed pathological changes in the lung tissues of phage-treated and control mice; results are shown in Figure 2. We observed that 24 h after *P. aeruginosa* inhalation, a large number of bacteria were present in the lungs of the control mice (Figure 2B). The alveoli were filled with red blood cells and mucus, and there was a tendency for severe pulmonary bleeding. Inflammatory cell infiltration in the lung interstitium was also observed. In contrast, in the lungs of treated mice, *P. aeruginosa* D4 was observed to be cleared, the observed bleeding tendency was mild, and only a small amount of RBCs were left in the pulmonary interstitium (Figure 2D). This indicates that KPP10 effectively eliminated *P. aeruginosa* D4 in the lungs.

### Number of Viable Bacteria and Phage in Lung and Serum

**FIGURE 3:**
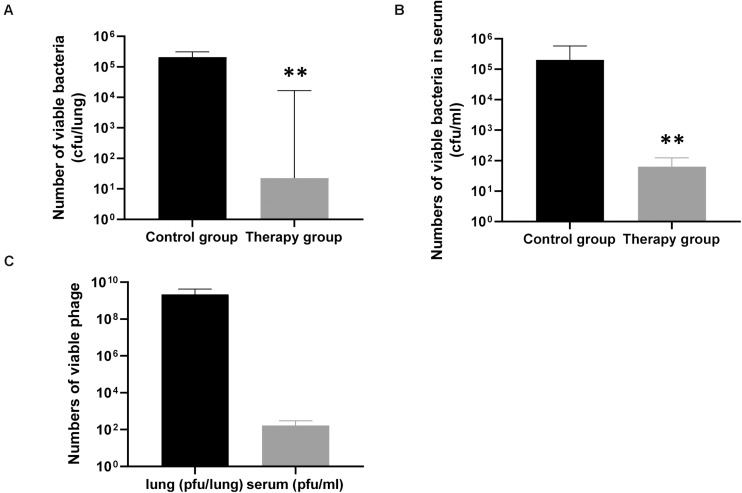
The number of viable bacteria and phage in the lungs and serum of mice 24 h after infection. The number of viable bacteria in the lungs **(A)**, the phage treatment group (*n* = 6) was significantly lower than that of the control group (*n* = 6). The number of viable bacteria in the serum **(B)**, the phage treatment group (*n* = 6) was also significantly lower than that of the control group (*n* = 6). Data are presented as mean ± SEM of two experiments. The number of viable phages in the lungs and serum **(C)** of phage-treated mice 24 h after infection. Data are presented as mean ± SEM of two experiments (** < 0.01 vs. untreated control). CFU denotes colony forming unit. PFU denotes plaque forming unit.

To confirm the effect of KPP10 on *P. aeruginosa* D4 in the lungs, we counted the viable bacteria and phage in the mouse lungs and serum. As shown in Figure 3, the number of viable bacteria in the lungs of the phage treatment group was significantly lower than that of the control group (treatment group 3,165 CFU/lung vs. control group 227,250 CFU/lung; *p* < 0.01) (Figure 3A), and the number of viable bacteria in the serum of the phage treatment group was also significantly lower than that of the control group (treatment group 64 CFU/mL vs. control group 202,111 CFU/mL; *p* < 0.01) (Figure 3B). This indicates that the *P. aeruginosa* D4 was significantly cleared from lungs and blood of the mice at 24 h post inhalation of KPP10. On the contrary, a large number of viable phages were persisting in the lungs in phage treatment group (2,217,833,333 PFU/lung), and the viable phages were also detected in serum (170 PFU/mL) (Figure 3C).

A question could be raised that bacteriophage came in contact with bacteria during harvesting and platting procedures and it could be worth if bacteria is separated from phage or phage activity is blocked by any means before culturing the bacteria in lungs and serum. A number of past studies (Watanabe et al., 2007; Mai et al., 2015; Hua et al., 2018) showed phage efficacy, in particularly phage KPP10 efficacy, in infection models describing the correlation between survival and bacterial numbers in circulation and in different organs in infected and control models. In our study, we followed the same method to demonstrate the efficacy of KPP10 in Pa infection models. In addition, we kept the collected samples on ice during all steps from the harvesting to culturing, which should reduce the activities of bacteria and phage to a minimum level without affecting their viability.

### Cytokine Levels in Serum and Lung Tissue Homogenate Samples

**FIGURE 4:**
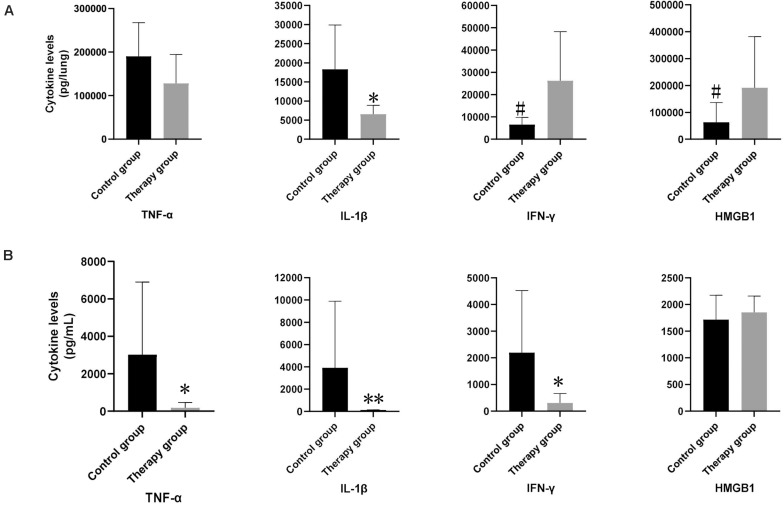
The levels of inflammatory cytokines in mouse lungs and serum. In lung samples **(A)**, the levels of IL-1β in phage-treated mice (*n* = 6) were significantly lower than those in control mice (*n* = 6). In serum samples **(B)**, the levels of TNF-α, IL-1β and IFN-γ in phage-treated group were significantly lower than that of the control group, however, HMGB1 had no significant difference. Data are presented as mean ± SEM of two experiments (* < 0.05, ** < 0.01 vs. untreated control).

We measured the levels of inflammatory cytokines TNFα, IL-1β, IFN-γ, and HMGB1 in the mice serum and lung homogenates. In the serum samples, levels of TNFα, IL-1β and IFN-γ in the phage-treated mice were significantly lower than those in the control mice (TNFα in treatment group: 185.82 pg/mL vs. control group: 3,026.24 pg/mL; *p* < 0.05; IL-1β in treatment group: 130.60 pg/mL vs. control group: 3,910.77 pg/m; *p* < 0.01; IFN-γ in treatment group: 307.02 pg/mL vs. control group: 2,192.05 pg/mL; *p* < 0.05) (Figure 4B). There was no significant difference observed in HMGB1. In the lung samples after tissue homogenization, the IL-1β level of the phage-treated group was significantly lower than that of the control group (treatment group: 2,179.33 pg/lung vs. control group 6,099.86 pg/lung; *p* < 0.05), TNFα level showed a decreasing tendency, whereas IFN-γ and HMGB1 showed an increasing tendency (Figure 4A).

### Endotoxin Levels of Serum Samples

**FIGURE 5:**
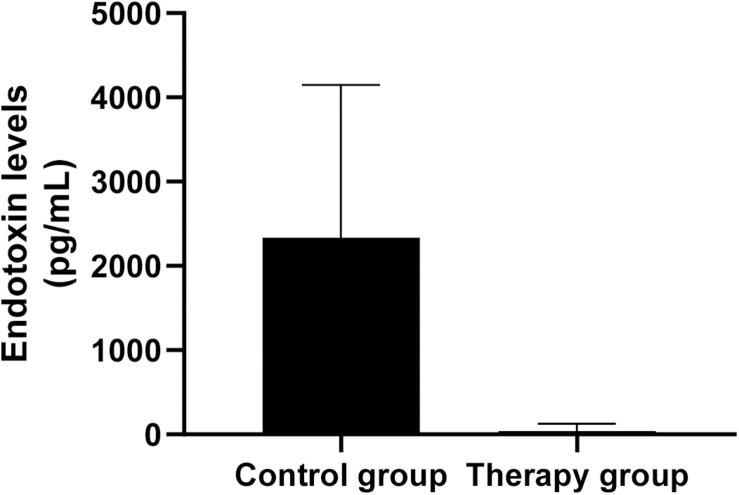
Endotoxin levels in mouse serum. The level of endotoxin in the serum of phage-treated mice (*n* = 6) was significantly lower than that of the control mice (*n* = 6). Data are presented as mean ± SEM of two experiments (** < 0.01 vs. untreated control).

To assess whether *P. aeruginosa* D4-induced pneumonia treated with KPP10 may introduce a risk of endotoxic shock, we measured the endotoxin levels in the serum of mice. As shown in Figure 5, endotoxin levels in the serum of the phage treatment group were significantly lower than those of the control group (treatment group: 38.51 pg/mL vs. control group: 2,337.03 pg/mL; *p* < 0.01). The decreased endotoxin (LPS) level in phage treated mice reflects the lower number of bacteria in mice circulation, which also correlate with the data of bacterial burden in phage-treated and non-treated mice in this study.

## Discussion

*Pseudomonas aeruginosa* is an important opportunistic pathogen, and its strong adaptability enables it to survive in various environments including surfaces of medical devices (Gellatly and Hancock, 2013). This makes *P. aeruginosa* one of the common pathogens causing pneumonia in patients relying on mechanical ventilation (Parker et al., 2008). *P. aeruginosa* has strong natural resistance to antibiotics, and its acquired antibiotic resistance is also gradually increasing (Berube et al., 2016). In terms of the need for new antibiotics due to increase in antibiotic resistance, the World Health Organization has classified *P. aeruginosa* under Priority 1 (Critical), which means that the establishment of phage therapy as a treatment modality that can replace antibiotic therapy is urgent [Viertel et al., 2014; World Health Organization (WHO), 2013]. Here, we showed that phage therapy is comparable with antimicrobial therapy for treating *P. aeruginosa* pneumonia.

Decrement of bacterial load in infected host due to bactericidal effect of phage is a known phenomenon (Rodriguez-Gonzalez et al., 2020). In this study, quantification of live bacteria and phage in the lungs and serum and, pathological changes in lung fields in experimental models are corresponding to the phenomenon. Moreover, we observed that the pneumonic mice those didn’t receive phage therapy mostly died within 24 h post infection. This finding supports the notion that phage replication rate in suitable bacteria is very high and it causes lysis of the infected bacteria within several hours (Payne and Jansen, 2001). Our study evident that the early administration of phage (at 2 h post infection) saved more infected mice than that of later administration at 8 h post infection. It is noticeable that the phage level in the lungs of the phage-treated mice was very high at 24 h p.i. It can be explained by the well-known characteristics of phage, that it releases progeny bacteriophages during lysis of bacteria. We have to admit that nasal inhalation of bacteria and bacteriophages cannot guarantee their even distribution in the lungs and multiple bacteriophages may infect the same bacteria due to the very high MOI. The number of bacteriophages in the area with low MOI will increase while the number of phages in the area with a higher MOI will decrease. This is the cause why phage level in the lungs of the mice in the treatment group remained high or basically unchanged at 24 h p.i.

Although previous many studies made pneumonia models in their experimental animals using inhalation of bacteria, but didn’t consider or describe the development of pulmonary septicemia in their models (Debarbieux et al., 2010; Forti et al., 2018). Here in our study, we explored the presence of bacteria and bacteriophage in blood, indicating that phage inhalation is not only beneficial to protect pulmonary infections but also to manage the septicemia triggered by pulmonary infections.

There is evidence that when a local acute bacterial infection occurs, T cells become activated and infiltrate the infected site (Sadikot et al., 2005). Infiltrating T cells produce IFN-γ to enhance phagocytic cells, especially polymorphonuclear neutrophils (PMN), to combat bacterial infection (Wagner et al., 2008). This may explain why phage-treated mice had high levels of IFN-γ in the lungs and very low levels of IFN-γ in the serum. We believe that this is beneficial in combating *P. aeruginosa*-mediated lung infections.

HMGB1 is considered to be the lethal endogenous mediator of endotoxins in mice. HMGB1 can be produced under the stimulation of endotoxins, TNFα, and IL-1β (Martinotti et al., 2015); moreover, recent studies have shown that cell damage also leads to HMGB1 expression (Kang et al., 2014). The levels of endotoxin, TNFα, and IL-1β in the phage treatment group were significantly lower than those in the control group, but the level of HMGB1 were higher than that of the control group. This suggests that treatment with KPP10 may induce cell damage. Here, we did not include experiments that would exclude the effects of cell damage, which is a limitation of our study.

Since 1900, phage therapy has been widely studied as treatment against bacterial infections. Phage therapy has many advantages: it has bactericidal properties, is self-generating, has little effect on normal microflora, is also effective against antibiotic-resistant bacteria, and seems to be able to destroy bacterial biofilms (Harper et al., 2014); however, controversy still exists regarding safety of phage therapy. While phages do not infect human cells, the endotoxin released by the phage associated Gram negative bacteria during bactericidal process of phage may trigger endotoxic shock in patients (Dickson and Lehmann, 2019). Phage therapy is currently used to treat skin infections and persistent lung infections in patients with cystic fibrosis (Krylov et al., 2016; Chang et al., 2020). The safety of phage therapy for treatment of acute lung infections caused by gram-negative bacteria needs further study, though previous studies have shown that treatment with bacteriophage strain KPP10 can protect mice from intestinal septicemia caused by *P. aeruginosa* D4 (Matsumoto et al., 1997a,b, 1998a,b,c; Watanabe et al., 2007). We find that inhaled KPP10 also exerts a significant protective effect against pneumonia caused by *P. aeruginosa* D4.

The additional advantages of KPP10 bacteriophage and *P. aeruginosa* D4 strain are that the detail genomic data of these elements are readily available. No study investigated KPP10 against *P. aeruginosa* pneumonia before ours, although many phages have been studied in the past. We noticed that in some studies those used the same animal models as ours, the survival rate of the phage treatment group did not reach 100%, which can often be achieved by antibiotic therapy (Hua et al., 2018; Jeon and Yong, 2019). However, bacteriological data showed that the level of bacteria in the lung was significantly reduced. Therefore, we are concerned that phage therapy has the risk of causing endotoxic shock. Considering effectiveness and safety of phage KPP10 revealed by this study, phage therapy may be useful in treating lung *P. aeruginosa* infections. Further research should be conducted to elucidate the safety of phage therapy and the possibility of it being used as an alternative treatment in clinical settings.

## Data Availability Statement

The raw data supporting the conclusions of this article will be made available by the authors, without undue reservation.

## Ethics Statement

The animal study was reviewed and approved by Tokyo Medical University Animal Care and Use Committee.

## Author Contributions

SN and TM: conception and design of the study, interpretation of data, and review the article. XY and AH: analysis and interpretation of data, collection and assembly of data, and drafting of the article. SM: phage preparation. All authors contributed to the article and approved the submitted version.

## Conflict of Interest

The authors declare that the research was conducted in the absence of any commercial or financial relationships that could be construed as a potential conflict of interest.
